# A systematic analysis of China's noncommunicable disease policies: policy themes, policy instruments, and policy effectiveness from 2009 to 2025

**DOI:** 10.3389/fpubh.2025.1643400

**Published:** 2025-09-18

**Authors:** Defeng Dong, Haojun Huang, Aitong Zhou, Chen Dong

**Affiliations:** ^1^School of Sport Management, Shandong Sport University, Jinan, China; ^2^School of Nursing, Yanbian University, Yanji, China; ^3^School of Management, Beijing Sport University, Beijing, China

**Keywords:** chronic disease/prevention and control, health policy, public health, policy making, content analysis

## Abstract

**Background:**

Noncommunicable diseases (NCDs) have become a leading threat to life and health. China is facing a more severe challenge from NCDs than ever before, which not only places enormous pressure on the public health system but also significantly hinders economic development and the achievement of sustainable development goals.

**Objective:**

Scientifically sound policies can provide a supportive environment for the prevention and control of NCDs. Therefore, this study aims to identify the strengths and weaknesses of current policies through a multidimensional analysis of policy texts, and to propose corresponding policy recommendations. The goal is to enhance the government's capacity and effectiveness in NCD governance, improve the health level of the entire society, and provide policy insights for China and other countries suffering from NCD burdens.

**Methods:**

This study adopts the Latent Dirichlet Allocation (LDA) topic model, content analysis method, and the Policy Modeling Consistency (PMC) model to conduct a multidimensional evaluation of 50 NCD-related policy documents issued by the Chinese government from 2009 to 2025, focusing on three dimensions: policy themes, policy instruments, and policy effectiveness.

**Results:**

(1) LDA analysis identified six key policy themes: primary healthcare, health promotion, health equity, healthcare reform and regulation, NCD prevention and control, and public health governance. (2) There was a clear structural imbalance in the use of the three types of policy instruments: supply-type (49.1%), environment-type (36.4%), and demand-type (14.4%). (3) The PMC model was applied to 9 representative policies. Among these, 4 were rated “excellent” and 5 “acceptable,” with an average PMC score of 6.19, indicating moderate internal consistency and scientific design, yet with considerable variance in quality across policies.

**Conclusion:**

China's NCD policy framework shows generally sound structure and intent. However, imbalances in tool application, limited demand-side engagement, and insufficient cross-sectoral coordination remain challenges. Enhancing interdisciplinary integration, strengthening incentives, and promoting stakeholder collaboration are essential for optimizing NCD governance.

## 1 Introduction

With the intensification of population aging and changes in lifestyle, noncommunicable diseases (NCDs) have become the leading causes of morbidity, disability, and mortality (1–3), posing serious threats to human health and imposing a substantial socio-economic burden on nations (1). The World Health Organization (WHO) defines it as: Noncommunicable diseases (NCDs), also known as chronic diseases, tend to be of long duration and are the result of a combination of genetic, physiological, environmental and behavioral factors. Major types include cardiovascular diseases, cancers, chronic respiratory diseases, and diabetes (4). In the *2030 Agenda for Sustainable Development* adopted at the United Nations Sustainable Development Summit in 2015, NCDs were recognized as a major public health challenge (5). Countries around the world have set ambitious goals and implemented various intervention measures to effectively manage NCDs. However, a decade later, the WHO reported in its World Health Statistics 2025, released in May 2025, that nearly all countries and regions are unable to achieve the target of reducing premature mortality from NCDs by one-third through prevention and treatment by 2030, as outlined in Sustainable Development Goal 3.4 (part of the Sustainable Development Goals (SDGs)). This goal is particularly difficult to achieve in low- and middle-income countries (6, 7). This poses a serious barrier to the sustainable socio-economic development and the promotion of health equity, and is likely to further widen the health and economic disparities between developing and developed countries.

As the largest developing country and the second most populous nation in the world, China is facing an especially severe challenge from NCDs. In 2021, at least 43 million deaths worldwide were attributed to NCDs, accounting for 75% of all non-pandemic-related deaths globally. In China, the situation is even more severe, with 91% of deaths and 86.7% of disability attributed to NCDs (4, 8, 9), Major NCDs contribute to nearly 90% of China's total health-related expenditures-a burden that continues to grow (9). Research and statistical data indicate that China has become the country with the most severe population aging and the largest number of individuals who are overweight or obese in the world (10, 11), These two groups are considered high-risk populations for NCDs, suggesting that China will face an even more formidable challenge in NCD prevention and control in the coming years than ever before (12).

The *Adelaide Statement on Health in All Policies* emphasizes that sound public policies can create supportive environments that enable people to lead healthy lives (13). The development of NCDs is influenced by a complex interplay of social, economic, environmental, and metabolic factors. Public health policies play an indispensable role in NCD management, as proactive intervention and management measures can effectively prevent and control these conditions (4). To address the growing burden of NCDs, alleviate pressure on the public health system, and promote population health, the Chinese government has issued several key policies, including the *Healthy China 2030 Planning Outline and the National Plan for NCD Prevention and Control*. These policies emphasize the integration of health into all policies and aim to establish a comprehensive policy support system for NCDs across the full process and life course, covering prevention, treatment, and rehabilitation (14).

International academic research on NCD policy is extensive, encompassing both qualitative and quantitative approaches. Draper et al. evaluated the consistency between NCD policy and practice using a case study method and found that significant gaps existed between policy and implementation. These gaps were primarily due to limited awareness of the policy among primary care physicians and insufficient community infrastructure (15). Numerous scholars have conducted extensive research on approaches to alleviating the economic burden on patients with NCDs. These approaches include alternative payment models (16), the integration of medical care and elderly services (17), health insurance schemes (18), transfer payments (19), community-based interventions (20), and patient self-management (21, 22), Such research has yielded valuable recommendations to reduce patient burden. Scholars have also examined NCD governance from both a policy design and assessment perspective (23). In terms of design, researchers have explored evidence-based decision-making (24, 25), policy pilot expansion, and intersectoral collaboration (26–28). Regarding evaluation, methods such as content analysis (29), comparative assessment (30), and difference-in-differences have been applied to analyze related documents (31). Notably, the mechanisms and impacts of four major risk factors–unhealthy diets, physical inactivity, smoking, and harmful use of alcohol–on NCDs have become a research hotspot, promoting interdisciplinary collaboration across public administration, sports science, medicine, behavioral science, and nutrition (32).

Although China's NCD policy has achieved certain outcomes, the lack of evidence-based decision-making has led to poor feasibility in some policies (33). In China, disparities in regional and urban-rural healthcare service capacity are major factors affecting health equity (14). In addition, an imbalanced population age structure, the digital divide caused by the rapid development of digital technologies, and differences in individuals' ability to afford NCD-related services also hinder the realization of health equity (34), The Chinese government provides medical insurance coverage to 95% of the population through the Urban Employee Basic Medical Insurance, the Urban-Rural Resident Basic Medical Insurance, and medical assistance for low-income households. However, the rapid increase in the NCD population poses significant challenges to the public health system, indicating a need for further optimization of the insurance system (35). Effective policy implementation depends on governmental leadership and interdepartmental coordination (34). In China, bureaucratic fragmentation and inter-agency competition have become key barriers to the effectiveness of collaborative NCD governance. To explore the underlying causes of these issues and seek viable solutions, Chinese scholars have extensively studied NCD management in impoverished areas, NCD diagnosis and treatment mechanisms, influencing factors in policy implementation, and healthcare security policy (36, 37). Among these studies, a growing number of scholars have adopted a policy instruments perspective, conducting content analyses of policy texts by constructing policy analysis frameworks (8, 38). Although several studies have examined China's NCD policy framework, many rely predominantly on qualitative methods, which weakens result reliability. Additionally, narrow analytical perspectives limit systematic evaluation. In response, this study adopts a comprehensive analytical approach using the Latent Dirichlet Allocation (LDA) topic model, content analysis, and the Policy Modeling Consistency (PMC) model to examine China's NCD policy texts from three dimensions: policy themes, policy instruments, and policy effectiveness. This study aims to answer: What are the main themes of current NCD governance? Are the policy instruments appropriately applied? How effective is the existing framework? This study contributes to further improving the NCD policy system in China and provides a useful reference for optimizing NCD policy in other countries, particularly in developing nations.

## 2 Materials and methods

### 2.1 Data collection

To ensure the validity and reliability of this study, NCD-related policy documents were retrieved using keywords such as “chronic disease” and “noncommunicable disease” from official government websites, including Peking University Law Database, the State Council, the National Health Commission, and the National Development and Reform Commission. The time range for the search is from January 1, 2009, to January 1, 2025. The types of policy documents searched include official announcements, laws and regulations, strategic plans, key work points, guiding opinions, and relevant industry guidelines, all of which have been formulated and publicly released by authoritative institutions, ensuring the authority and credibility of the policy research sample (39). The year 2009 was selected as the starting point for data collection because the Chinese government released the *Opinions on Deepening the Reform of the Medical and Healthcare System* that year, which marked the launch of the “new medical reform” initiative. This reform emphasized that the government bears the primary responsibility for ensuring access to healthcare services (40), and advocated for a prevention-oriented approach, integrating health into all aspects of healthcare services. It also reflected a paradigm shift from a treatment-centered to a health-centered healthcare system. The policy set a national goal of safeguarding the health of all citizens and laid a solid institutional and policy foundation for the systematic development and implementation of NCD policy. The entire search was conducted by two professionals engaged in policy research, and the search work was completed by February 2025. A total of 936 NCD policies were identified. The Chinese government websites generally update policy documents in a timely manner, ensuring that we were able to access the most recently released relevant policies. To further ensure the comprehensiveness and authority of the policies, the collection and screening of policy texts were carried out by three researchers with policy research backgrounds, followed by multiple rounds of cross-checking to fill any gaps.

### 2.2 Data screening

The screening of policy samples was based on the principles of authority, relevance, and validity. The specific criteria were as follows: (1) Authority: Policy documents included in the study were those classified as administrative regulations, departmental rules, or departmental normative documents that had been officially issued by authoritative national institutions. Given that most local policies in China are developed based on national-level policies, this study excluded local policies from the sample (41). (2) Relevance: Only policy documents with high relevance to the theme of this study–noncommunicable diseases (NCDs)–were included. Documents such as meeting notices and training announcements, which were only marginally related, were excluded (41). (3) Validity: Only currently valid policies were included in the sample; expired policies were not considered. To ensure the rigor and accuracy of the selection process, three experienced policy researchers independently reviewed the policy texts. In cases of disagreement, the inclusion decision was made after discussion with subject matter experts. Based on these criteria, a total of 50 NCD policy documents were retained after two rounds of screening (Table 1).

**Table 1:** Sample library of NCD policy documents (partial list).

**Item**	**Policy name**	**Date issued**
1	Opinions of the CPC Central Committee and the State Council on Deepening the Reform of the Medical and Healthcare System	March 17, 2009
2	Opinions on Promoting the Gradual Equalization of Basic Public Health Services	July 9, 2009
3	Guidelines for the Implementation of Comprehensive Prevention and Control Demonstration Zones for Chronic Non-communicable Diseases	November 8, 2010
4	National Standards for the Prevention and Control of Chronic Diseases	March 3, 2011
…	…	…
25	National Nursing Development Plan (2016–2020)	November 18, 2016
26	The 13th Five-Year Plan for Health and Wellness	December 27, 2016
27	The 13th Five-Year National Plan for Health Promotion and Education	January 11, 2017
28	China's Mid- and Long-Term Plan for the Prevention and Control of Chronic Diseases (2017-2025)	January 22, 2017
…	…	…
47	Opinions on Further Deepening Reform to Promote the Healthy Development of the Rural Medical and Healthcare System	February 23, 2023
48	Implementation Plan for the “Weight Management Year” Campaign	June 6, 2024
49	Detailed Rules for Food Safety and Nutrition and Health Work in Disease Prevention and Control Institutions	August 19, 2024
50	Guiding Opinions on Promoting the High-Quality Development of Integrated Medical and Elderly Care Services	December 12, 2024

### 2.3 Research procedure and design

Policy themes, policy instruments, and policy effectiveness are all critical components of public policy analysis (42). Policy themes reflect the core content of a policy and serve as a concentrated expression of government priorities, policy objectives, and future development trends (43). Policy instruments refer to the measures adopted by policy actors to achieve policy objectives and act as the bridge between policy goals and policy outcomes (44, 45). Policy effectiveness refers to the impact and outcomes of a policy in achieving its intended goals. It includes both content effectiveness and implementation effectiveness, serving as a direct indicator of policy validity and forming the basis of policy design (42, 46). There exists an interrelated and interactive mechanism among policy themes, instruments, and effectiveness (47). Under the guidance of clearly defined policy themes, when instruments are reasonably balanced and appropriately aligned with policy objectives, the policy tends to exhibit a higher level of effectiveness (43).

**Figure 1:**
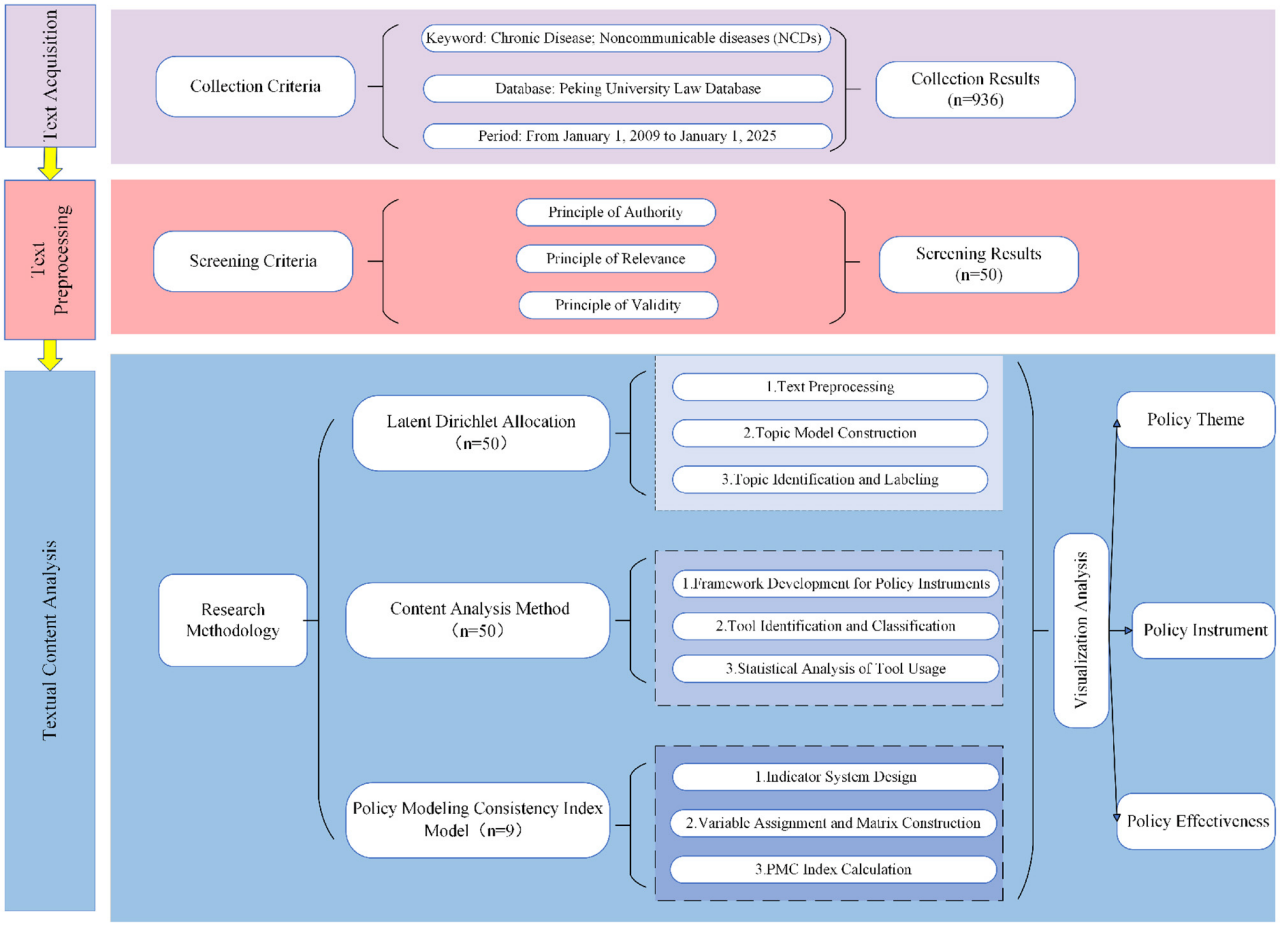
Research design.

To systematically explore the principles and mechanisms underlying the formulation of NCD policy in China, and to assess the overall quality and expected outcomes of policy design, this study adopts a mixed-methods approach that integrates both qualitative and quantitative techniques. A three-dimensional analytical framework—comprising policy themes, policy instruments, and policy effectiveness—was developed to analyze NCD policy from multiple perspectives (Figure 1). Specifically, in the policy theme dimension, the Latent Dirichlet Allocation (LDA) topic model was employed to perform topic modeling and identify key policy themes (*n*= 50). In the policy instrument dimension, content analysis was conducted based on policy instrument theory to examine the use of instruments (*n*=50). To ensure the relevance, depth, and comparability of the policy evaluation results, and in view of the PMC-index model's suitability and efficiency in small-sample policy assessment (48), this study selected nine representative NCD-related policy documents from the sample pool (*n*=50). These policies were chosen based on their issuance date, issuing authority, document type, and thematic relevance, and served as the modeling samples for the policy effectiveness dimension (Table 2).

**Table 2:** Policy sample library evaluated using the PMC model.

**Item**	**Policy name**	**Date issued**
P1	Guidelines for the establishment of demonstration zones for comprehensive Prevention and control of non-communicable chronic diseases	November 8, 2010
P2	National standards for the prevention and control of chronic diseases	March 3, 2011
P3	China national plan for the prevention and control of chronic diseases (2012–2015)	May 8, 2012
P4	Work plan for chronic disease and nutrition surveillance among Chinese residents	September 10, 2014
P5	China's Three-Year Action Plan for Cancer Prevention and Control (2015–2017)	September 9, 2015
P6	Measures for the construction and management of national demonstration zones for comprehensive prevention and control of chronic diseases	October 20, 2016
P7	China's mid- and long-term plan for the prevention and control of chronic diseases (2017–2025)	January 22, 2017
P8	Work plan for family doctor contracted services for registered poor populations with chronic diseases	July 12, 2018
P9	Implementation plan for the “weight management year” campaign	June 6, 2024

This framework begins with LDA topic modeling to identify and understand the main content of policies and employs qualitative methods to extract and synthesize key policy themes. Subsequently, based on policy instrument theory, content analysis is used to reveal the application of policy instruments. Finally, combining the findings from the theme analysis and instrument analysis, a quantitative evaluation model is constructed to assess the content effectiveness of NCD policy documents. By integrating multiple research methods, progressing across analytical layers, and combining different perspectives, this framework addresses the limitations inherent in single-method approaches and enhances the scientific rigor, systematic structure, and comprehensiveness of the research (48, 49). Moreover, through the use of visual representation, the framework improves the readability of research findings and facilitates the dissemination of results.

### 2.4 Research methods

#### 2.4.1 Latent Dirichlet allocation (LDA) topic model

The LDA topic model is a statistical language model proposed by Blei (50), based on a three-layer Bayesian probabilistic framework (word-topic-document) (51). This model uses unsupervised machine learning to extract latent thematic structures from large volumes of unstructured text data (52), thereby reducing biases caused by subjective interpretation and improving the accuracy and scientific rigor of research outcomes (50, 53) As a machine-learning-assisted qualitative method, the LDA model is widely applied in text analysis research (54), such as in the analysis of news reports and public opinion (50, 54, 55). Accordingly, this study applies the LDA topic model to extract and analyze policy themes from 50 NCD policy documents in the sample library. The joint probability distribution of the LDA model reflects the probabilistic relationship among documents, topics, and words, as shown in Equation 1.


(1) $$\begin{array}{l} P\left(d,w,z,\theta ,\varphi \right)=P\left(\theta |\alpha \right)\times \prod P\left(z|\theta \right)\times P\left(w|z,\varphi \right)\times \prod P\left(\varphi |\beta \right) \end{array}$$

where the word ω in document *d* is associated with topic *z*; θ represents the topic distribution within the document, and φ denotes the word distribution within each topic. α and β are hyperparameters that control the prior distributions of θ and φ, respectively.

**Figure 2:**
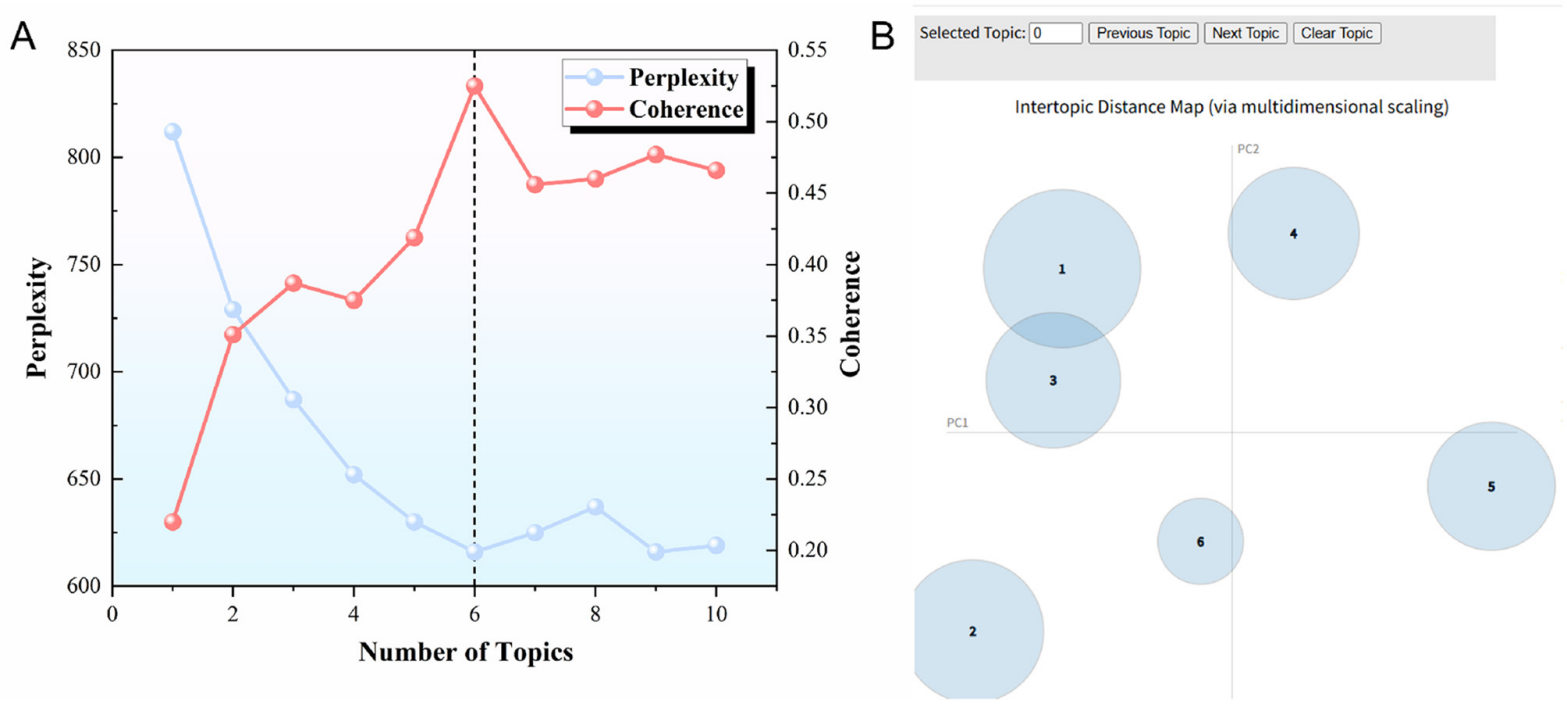
**(A)** Perplexity and coherence score curves; **(B)** Topic distribution bubble chart. **(A)** Shows the relationship between the number of topics and the perplexity (blue line) and coherence (red line) scores. When the number of topics is 6 (indicated by the dashed line), the model achieves a higher coherence level and lower perplexity, suggesting that 6 is the optimal number of topics for the model. **(B)** Visualizes the distances between topics using multidimensional scaling, with each circle representing a topic. The size of the circle corresponds to the importance of the topic, and the axes (PC1, PC2) reflect the relationships between topics based on the model's output.

The specific modeling process consisted of the following steps: (1) Based on a thorough reading of the policy documents, the text was preprocessed by merging synonyms, removing irrelevant words, and standardizing synonymous terms. A domain-specific vocabulary for NCD policy texts was created, and a Bag of Words model was constructed (56). (2) LDA topic modeling was conducted using the Python-based Gensim library. The Coherence Model and LDA Model modules in Gensim were used, with the hyperparameters α and β set to 50/k and 0.1, respectively. The model was trained with 100 iterations. To ensure the robustness of the LDA model, perplexity and topic coherence scores were calculated based on Equations 2 and 3, respectively (Figure 2A) (57).


(2) $$\begin{array}{l} \text{Perplexity}=\exp\left(-\frac{\sum_{d\in D}\log P\left(w_{d}|\alpha ,\beta \right)}{\sum_{d\in D}N_{d}}\right) \end{array}$$

Where *P*(*w*_*d*_∣α, β) represents the marginal likelihood of document *d* given the hyperparameters α and β. A lower perplexity value indicates a better fit of the model to the test data.


(3) $$\begin{array}{l} \text{Coherenc}e_{\text{UMass}}=\frac{2}{N\left(N-1\right)}\overset{N}{\underset{i=2}{\sum }}\overset{i-1}{\underset{j=1}{\sum }}\log\left(\frac{D\left(w_{i},w_{j}\right)+\varepsilon }{D\left(w_{j}\right)}\right) \end{array}$$

Where *D*(*w*_*j*_) and *D*(*w*_*i*_, *w*_*j*_) denote the number of documents in the corpus containing word *w*_*j*_ and the co-occurrence of words *w*_*i*_ and *w*_*j*_, respectively. ε is a smoothing parameter, typically set to 1. A higher coherence score indicates stronger co-occurrence relationships among words within the topic and thus a higher level of semantic consistency.

As shown in Figure 2A, when the number of topics is set to six, the model exhibits relatively low perplexity and high coherence scores. Therefore, this study sets the number of LDA topics at six. Visualization was then performed using the PyLDAvis library, generating Figure 2B. It can be observed that the six topics are relatively distinct and dispersed across all four quadrants, indicating that each topic represents a key focus area in the NCD policy discourse. (3) Keywords under each topic were subsequently extracted, organized, and labeled with appropriate topic tags. The importance of each topic within the corpus was then calculated based on Equation (4), which reflects the distribution of topics across the entire text set.


(4) $$\begin{array}{l} {\text{Strength}}_{k}=\frac{1}{D}\overset{D}{\underset{d=1}{\sum }}P\left(z_{k}|d\right) \end{array}$$

where *P*(*Z*_*k*_∣*d*) represents the probability that document *d* belongs to topic *k*, and *D* denotes the total number of documents. A higher value of this indicator suggests that the topic holds greater importance within the NCD policy corpus.

#### 2.4.2 Content analysis method

Content analysis is a structured research method used to draw conclusions through systematic examination of textual content (2). Initially applied in the field of communication studies, the method was formally established as a scientific research tool with the publication of *Content Analysis in Communication Research* by American scholar Bernard Berelson in 1952 (58). As a non-intrusive analytical method, content analysis allows researchers to maintain neutrality during the analytical process and is particularly well-suited for handling large volumes of longitudinal data. As such, it has been widely applied in disciplines such as public administration, history, and other social sciences (59). In this study, NVivo 15 was employed to conduct content analysis on 50 NCD policy documents. The specific steps included constructing a policy instrument classification framework, coding and quantifying text data, and visualizing the results. The policy instrument framework was developed based on Rothwell's policy instrument theory. Unlike Howlett, who classifies policy tools according to their level of coerciveness into voluntary, coercive, and mixed tools (47), Rothwell categorizes them based on functional roles into supply-oriented, environment-oriented, and demand-oriented policy instruments (60). These categories reflect the distinct functional roles each type of instrument plays during policy implementation. In practice, supply-side and demand-side instruments exert direct push-and-pull effects, while environmental instruments offer indirect structural support. Specifically, supply-side instruments enable policy execution by providing critical resources such as funding, human capital, and technical infrastructure. Environmental instruments shape institutional and contextual conditions-such as laws, strategic plans, and incentive mechanisms-that indirectly facilitate policy implementation. Demand-side instruments play a mobilizing role by engaging target groups and non-governmental stakeholders through mechanisms like public-private partnerships, government procurement, and international collaboration. This study adopted Rothwell's classification and, in conjunction with the results of the LDA topic analysis and existing literature, identified subcategories for each type of instrument (Table 3) (36, 38).

**Table 3:** Types and definitions of policy instruments in NCD policy.

**Tool type**	**Tool name**	**Connotation**
Supply-side policy instruments	Public services	Health management for chronic disease populations and promotion of chronic disease prevention and control knowledge
	Talent development	Training professionals in chronic disease prevention and control
	Financial investment	Providing financial support for chronic disease prevention and control
	Infrastructure development	Constructing infrastructure for chronic disease prevention and control
	Information technology	Providing data and information support for chronic disease patients and healthcare institutions
	Standards and guidelines	Establishing chronic disease prevention and control guidelines and standardizing chronic disease diagnosis and treatment practices
Environmental policy instruments	Target planning	Developing Chronic disease prevention and control development plans
	Regulatory control	Strengthening legal constraints
	Strategic measures	Formulating a series of chronic disease prevention and control measures
	Financial and tax policies	Providing financial and tax incentives and support
	Supervision and Evaluation	Strengthening the supervision and evaluation of policy implementation
	Social Environment	Creating a positive social atmosphere for chronic disease prevention and control
Demand-side policy instruments	Government Procurement	Government purchase of chronic disease prevention and control medicines and services
	Public-private partnership	Collaboration between the government and social capital
	International exchange	Domestic and international academic exchange and cooperation
	Demonstration and pilot programs	Establishing chronic disease prevention and control demonstration zones
	Public welfare and charity	Encouraging public support for vulnerable populations

#### 2.4.3 Policy modeling consistency (PMC) index model

The PMC Index Model was proposed by Ruiz based on the Omnia Mobilis hypothesis, which holds that “everything in the world is broadly and closely interconnected.” It emphasizes that variable selection in policy modeling should be multi-dimensional and comprehensive, broadly considering relevant factors and avoiding the omission of any variable (61). This philosophy aligns with Howlett's perspective on policy analysis, which suggests that most complex policy issues cannot be addressed through a single tool alone, but instead require a coordinated mix of policy instruments supported by diverse governance resources (62). Compared with other evaluation approaches such as the entropy weight method or the analytic hierarchy process (AHP), the PMC Index Model more effectively reduces the influence of subjective judgments on research validity. Moreover, its modeling philosophy is consistent with the framework of this study and enhances the comprehensiveness and reliability of policy evaluation (48). The PMC Index calculation process includes variable selection and parameter identification, construction of a multi-input-output table, computation of the PMC index, and visualization of the quantitative results (63).

##### 2.4.3.1 Variable selection and parameter identification

In this study, variable selection was guided by Ruiz's modeling principle of “broadly considering relevant elements and omitting none” (64), as well as the results of the LDA topic modeling and policy instrument content analysis. Drawing upon existing literature and following a cross-blind selection process conducted by the research team, consensus was reached on a final set of nine primary variables and 51 secondary variables (Table 4).

**Table 4:** Evaluation index system of the PMC model for NCD policy.

**Primary variables**	**Secondary variables**
X1: policy nature	X1:1 regulation; X1:2 guidance; X1:3 description; X1:4 prediction; X1:5 diagnosis; X1:6 recommendation
X2: policy timeliness	X2:1 long-term (more than 5 years); X2:2 medium-term (1–5 years); X2:3 short-term (within 1 year)
X3: policy function	X3:1 coordination and cooperation; X3:2 rights protection; X3:3 institutional constraints; X3:4 standardized guidance; X3:5 supervision and regulation
X4: policy theme	X4:1 primary healthcare; X4:2 health promotion; X4:3 health equity; X4:4 healthcare reform and regulation; X4:5 chronic disease prevention and control; X4:6 public health governance
X5: policy evaluation	X5:1 clear objectives; X5:2 scientific plan; X5:3 detailed content; X5:4 sufficient basis
X6: policy audience	X6:1 government; X6:2 community; X6:3 schools; X6:4 enterprises; X6:5 chronic disease patients; X6:6 farmers; X6:7 children; X6:8 elderly; X6:9 disabled people; X6:10 social organizations
X7: policy tools	X7:1 environmental-type; X7:2 demand-type; X7:3 supply-type
X8: incentive measures	X8:1 talent incentives; X8:2 tax incentives; X8:3 financial support; X8:4 government subsidies; X8:5 demonstration projects
X9: related disciplines	X9:1 public health; X9:2 medicine; X9:3 health economics; X9:4 sociology; X9:5 psychology; X9:6 nutrition; X9:7 behavioral science; X9:8 environmental science; X9:9 law

##### 2.4.3.2 Construction of the multi-input-output table

The multi-input-output table is an optional analytical framework capable of accommodating large volumes of data and facilitating multidimensional quantitative analysis of policy texts (65). Following the modeling principles of the PMC Index Model, this study constructed a multi-input-output table for NCD policies (Table 5). To ensure equal weighting across all variables, a binary coding scheme was applied to quantify the presence of each secondary variable. Specifically, if a given secondary variable is addressed in the policy, it is assigned a value of 1; otherwise, it is assigned a value of 0 (26).

**Table 5:** Multi-input-output table for NCD policy.

**Primary variable**	**X1**	**X2**	**X3**	**X4**	**X5**	**X6**	**X7**	**X8**	**X9**
**Secondary variables**						X6:1			X9:1
	X1:1		X3:1	X4:1		X6:2			X9:2
	X1:2			X4:2	X5:1	X6:3		X8:1	X9:3
	X1:3	X2:1	X3:2	X4:3	X5:2	X6:4	X7:1	X8:2	X9:4
	X1:4	X2:2	X3:3	X4:4	X5:3	X6:5	X7:2	X8:3	X9:5
	X1:5	X2:3	X3:4	X4:5	X5:4	X6:6	X7:3	X8:4	X9:6
	X1:6		X3:5	X4:6		X6:7		X8:5	X9:7
						X6:8			X9:8
						X6:9			X9:9
						X6:10			

##### 2.4.3.3 Calculation of the PMC index

The specific steps for calculating the PMC Index are as follows: (1) assign binary values to each secondary variable according to Equations 5, 6; (2) calculate the value of each primary variable using Equation 7, where *t* denotes the primary variable, j represents the secondary variables, and *T* is the total number of secondary variables under each primary variable; and (3) sum the scores of all primary variables using Equation 8 to obtain the final PMC Index for each NCD policy (66). Furthermore, based on the calculated PMC Index values, the policies were rated using Estrada's classification criteria for policy levels (Table 6) (67).


(5) $$\begin{array}{l} X\sim N\left[0,1\right] \end{array}$$

(6) $$\begin{array}{l} X=\left\{XR:\left[0\sim 1\right]\right\} \end{array}$$

(7) $$\begin{array}{l} X_{t}\left(\overset{n}{\underset{j=1}{\sum }}\frac{X_{tj}}{T\left(X_{tj}\right)}\right),t=1,2,3,\cdots \infty \end{array}$$

(8) $$\begin{array}{c} \text{PMC}=\\ \left[\begin{array}{c} X_{1}\left(\sum_{i=1}^{6}\frac{X_{1i}}{6}\right)+X_{2}\left(\sum_{j=1}^{3}\frac{X_{2j}}{3}\right)+X_{3}\left(\sum_{k=1}^{5}\frac{X_{3k}}{5}\right)\\ +X_{4}\left(\sum_{l=1}^{6}\frac{X_{4l}}{6}\right)+X_{5}\left(\sum_{m=1}^{4}\frac{X_{4m}}{4}\right)+X_{6}\left(\sum_{n=1}^{10}\frac{X_{6n}}{10}\right)+\\ X_{7}\left(\sum_{o=1}^{3}\frac{X_{7o}}{3}\right)+X_{8}\left(\sum_{p=1}^{5}\frac{X_{8p}}{5}\right)+X_{9}\left(\sum_{q}^{9}\frac{X_{9q}}{9}\right) \end{array}\right] \end{array}$$

**Table 6:** Classification criteria for PMC index ratings of NCD policies.

PMC index	0–3.99	4–5.99	6–7.99	8–9
Rating	Poor	Acceptable	Excellent	Perfect

##### 2.4.3.4 Visualization of quantitative results

Visualizing the PMC index results facilitates a clear and intuitive understanding of how NCD policies perform across different variable dimensions. It enables a comprehensive and multidimensional representation of policy evaluation outcomes, aiding in the assessment of both overall strengths and weaknesses and specific indicator disparities (64). Based on the calculated PMC indices, this study generated PMC surface plots and radar charts that reflect the scores of the primary variables. The degree of convexity or concavity in the PMC surface illustrates the internal consistency and structural coherence of the policy text; a smoother surface implies a more comprehensive and coherent policy structure (68). The radar chart visually highlights areas of weakness across variable dimensions for each policy. The construction of a PMC matrix is a prerequisite for generating the PMC surface plot. In this study, a 3 × 3 matrix was constructed using the nine primary variables, as shown in Equation 9.


(9) $$\begin{array}{c} \text{PMC}=\left[\begin{array}{ccc} X_{1} & X_{2} & X_{3}\\ X_{4} & X_{5} & X_{6}\\ X_{7} & X_{8} & X_{9} \end{array}\right] \end{array}$$

## 3 Results and discussion

### 3.1 Analysis of external characteristics of policy texts

**Figure 3:**
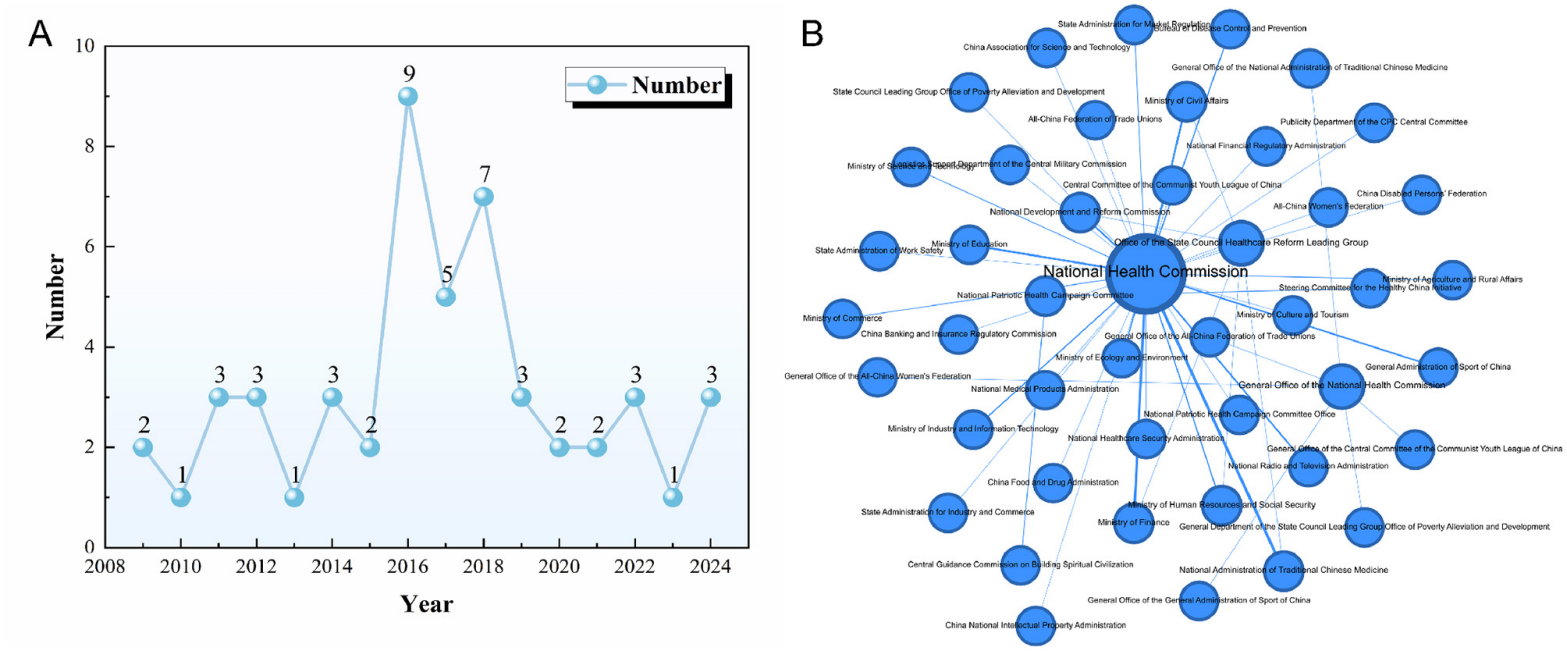
**(A)** Annual number of NCD policy documents issued in China (2009–2025); **(B)** Collaboration network of issuing agencies for NCD policies.

This study analyzed noncommunicable disease (NCD)-related policies issued by the Chinese government between 2009 and 2025. As shown in Figure 3A, the number of annual policy documents exhibited a relatively stable fluctuating trend. The years 2016, 2017, and 2018 saw a notable increase in policy issuance, with 9 NCD-related policies released in 2016 alone. This surge may be attributed to President Xi Jinping's 2016 call to “prioritize people's health in national development, emphasize prevention, and integrate health into all policies.” In August of the same year, the Chinese government launched the *Healthy China 2030 Planning Outline*, establishing “Healthy China” as a national strategy. Health promotion subsequently became a major focus of policy formulation. Regarding policy issuers, this study used Gephi software to generate a collaboration network of issuing agencies for NCD policies and to calculate inter-agency collaboration frequencies (Figure 3B). Among the 50 policy texts in the sample, 32 (64%) were issued by a single department, indicating insufficient interdepartmental collaboration in the policy formulation process. The State Council and the National Health Commission were the most frequent issuing bodies, with 16 and 14 policy issuances, respectively. According to the network analysis shown in Figure 3B, the National Health Commission occupied a central position in the collaboration network, with a degree centrality score of 34. This reflects the Commission's critical role within China's NCD policy system–not only as a primary policymaker but also as a central hub that facilitates coordination and integration across government departments.

### 3.2 Analysis of internal characteristics of policy texts

#### 3.2.1 Policy content theme analysis

**Figure 4:**
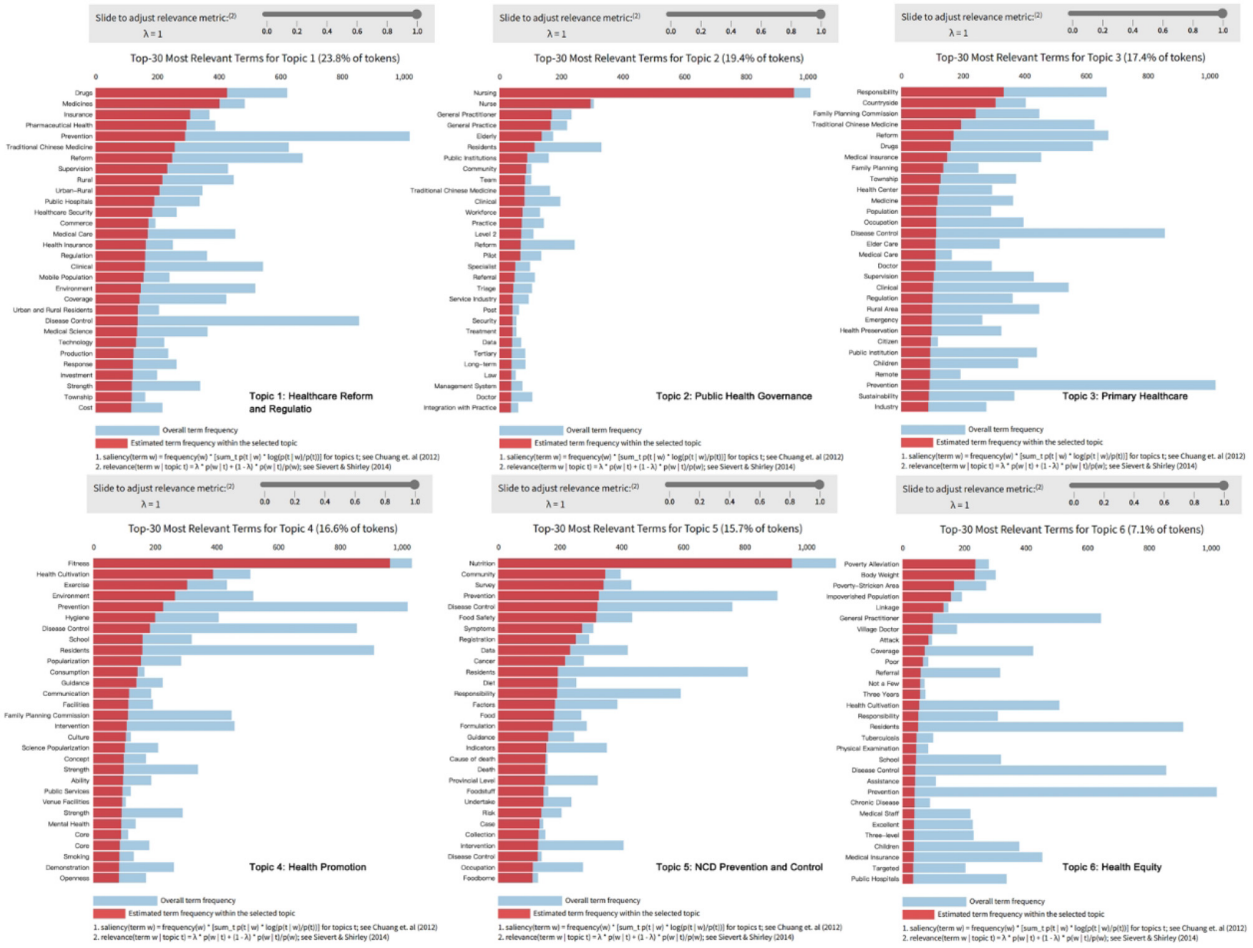
Summary of thematic dimensions in China's NCD policy framework. The charts display the overall term frequency for each topic (shown in blue) and the estimated term frequency for each topic (shown in red).

This study employs LDA topic modeling to analyze NCD policy texts. By selecting the top 30 high-frequency words most strongly associated with each topic, it extracts key terms and identifies six major policy themes: healthcare reform and regulation, public health governance, primary healthcare, health promotion, NCD prevention and control, and health equity (Figure 4). This comprehensively reflects the institutional logic and core strategies of the Chinese government in NCD governance.

##### 3.2.1.1 Healthcare reform and regulation

China's NCD policies focus on healthcare system reform and drug regulation, especially in the systematic adjustments of payment mechanisms and drug distribution governance. In the dimension of healthcare reform and regulation, policy texts frequently mention high-frequency words such as “drugs,” “medications,” “insurance,” “medical insurance,” “healthcare,” “reform,” and “regulation.” This reflects China's efforts to optimize payment mechanisms and resource allocation through a series of institutional regulations, while also using measures such as centralized bulk purchasing, medical insurance cost control, and payment reform to effectively reduce drug price margins. These measures aim to improve national healthcare efficiency and equity, and further enhance the full-process regulation of drug quality and usage safety.

##### 3.2.1.2 Public health governance

The NCD policy system in China demonstrates a trend of transformation toward collaboration and integration. High-frequency words in the theme of public health governance, such as “nursing,” “family doctor,” “general practice,” “referral,” and “tiered” indicate that NCD governance is moving toward a governance model of service integration and responsibility reconstruction. The policy focuses on resource coordination, organizational integration, and functional collaboration, gradually integrating primary healthcare institutions and the public health service system into a unified platform.

##### 3.2.1.3 Primary healthcare

The primary healthcare service system is key to advancing nationwide NCD governance. Currently, primary healthcare faces issues such as unequal resource distribution, a shortage of technology and talent, and weak information linkage mechanisms, resulting in disparities in health services between urban and rural areas, medical institutions, and regions (69). The high-frequency words in the figure, such as “village,” “health center,” “township,” and ”medical insurance,” indicate that the policy is focused on optimizing the primary healthcare structure, particularly in the fields of public health and disease prevention.

##### 3.2.1.4 Health promotion

With the continued deepening of the Healthy China strategy, health promotion has gradually shifted from a marginal promotional tool to one of the core approaches in NCD governance. In the current NCD policies, “fitness” and “exercise” are high-frequency terms. These terms indicate that the policy particularly emphasizes the popularization of group exercise and the promotion of public services in the field of health promotion. This policy trend reflects the government's efforts to promote nationwide participation in physical activities and the dissemination of healthy lifestyles through innovative policy measures and strengthening the construction of sports facilities. In addition, the current NCD policy texts also focus on health education, balanced diets, and mental health, gradually promoting the integration of health factors into various aspects of social development.

##### 3.2.1.5 NCD prevention and control

In this theme, high-frequency words such as “food safety,” “survey,” “death,” and “cancer” reflect the key points of NCD policy, emphasizing aspects such as nutritional intervention, disease screening, early warning, and the analysis of causes of death. The policy texts also focus on strengthening NCD prevention and control through information technology, with many policies mentioning the use of public data to build a monitoring system that includes nutritional risk identification, disease screening, and early warning, thereby advancing NCD prevention and control.

##### 3.2.1.6 Health equity

Health equity is a key component of China's NCD policy, with a focus on medical rights and healthcare security mechanisms for key populations such as the impoverished, those in remote areas, and the elderly. In the theme of health equity, high-frequency words such as “poverty alleviation,” “impoverished populations,” “poor areas,” and “responsibility” dominate, reflecting China's special focus on the two sub-dimensions of health equity and health poverty alleviation in NCD governance.

The six major themes of China's NCD policy reflect the construction of an integrated governance approach to NCDs, encompassing dimensions such as institutional design, prevention orientation, differential protection, payment systems, technical support, and grassroots integration. This demonstrates the systematic institutional design and hierarchical response of public policies with Chinese characteristics to social issues.

#### 3.2.2 Analysis of policy instruments

**Figure 5:**
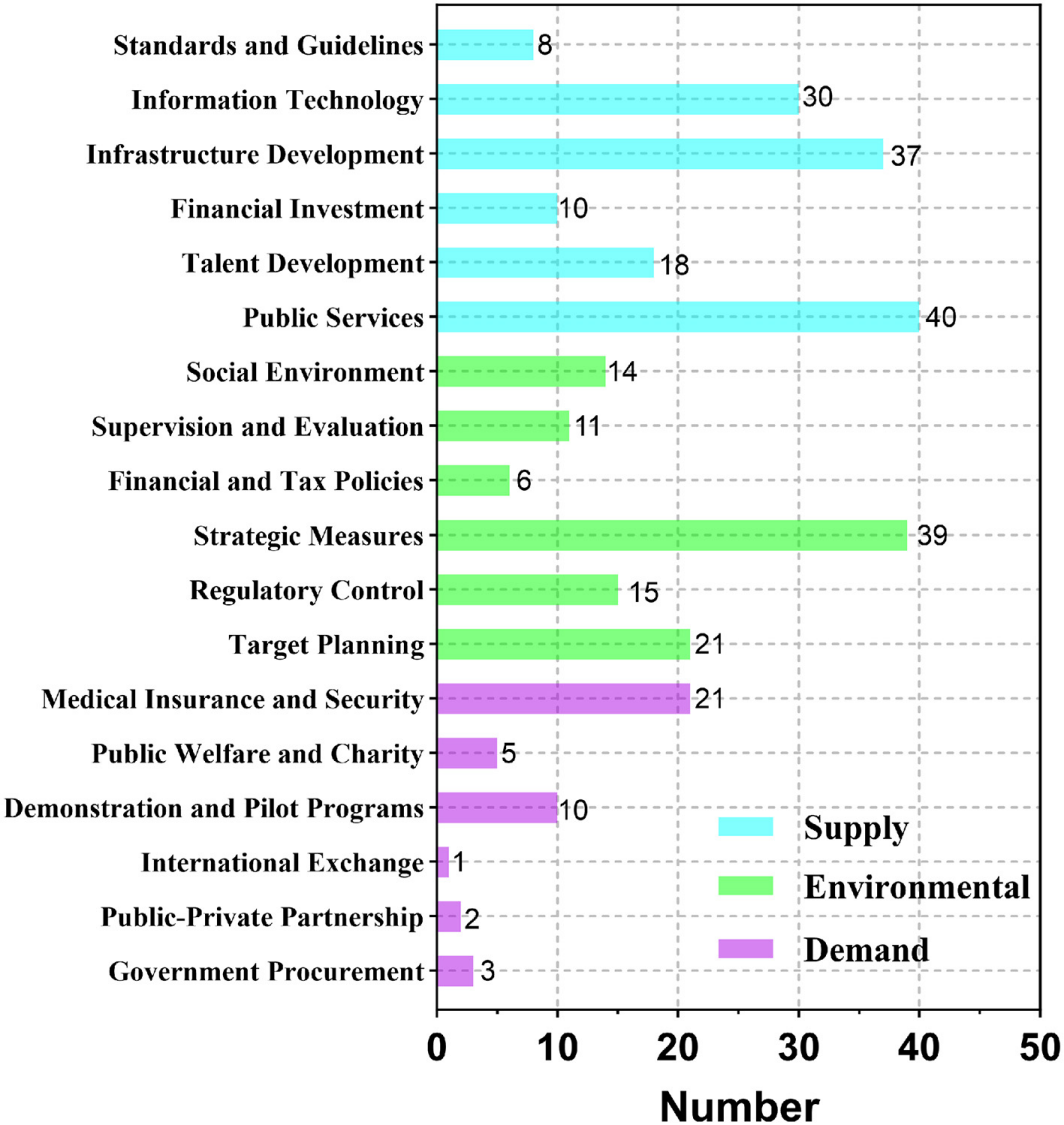
Utilization of policy instruments in China's NCD Policies. Frequency distribution of policy instruments across 50 NCD policy documents, categorized by type: supply-side (blue), environment-oriented (green), and demand-side (purple).

Based on the policy tools analysis framework and the coding of NCD policy texts, this study identified a total of 291 coding reference points for policy tools (see Figure 5). The coding results show significant differences in the use of policy tools within NCD policies. Specifically, supply-side, environmental, and demand-side policy tools account for 49.1% (143/291), 36.4% (106/291), and 14.4% (42/291), respectively. This indicates an imbalance in the use of policy tools within NCD policies, with a greater tendency for the government to use supply-side and environmental policy tools, while demand-side policy tools are used relatively less. This imbalance in the use of policy tools may limit the effectiveness of the policy tool combination, thereby affecting the comprehensive implementation and long-term advancement of the policies.

Among the supply-side policy tools, public services and infrastructure construction are the primary tools, ranking first and second among the 15 policy tools, accounting for 13.75% (40/291) and 12.71% (37/291), respectively. This indicates the government's emphasis on the construction of the primary healthcare service system. However, the three tools related to talent development, financial investment, and standardization together account for only 25.2% (36/143) of supply-side policy tools, suggesting that the use of policy tools for talent development and financial support is relatively limited, which restricts the sustainability and effectiveness of the policies.

Regarding environmental policy tools, while strategic measures account for 36.8% (39/106), reflecting the flexibility of policy design, there is significant discoordination in actual implementation. This may be due to insufficiently detailed policy content, leading to unclear responsibilities and accountability among policy implementers, which affects the effectiveness of policy execution. The financial incentives and supervision assessment tools account for 5.7% (6/106) and 10.4% (11/106), respectively. This suggests that the current policy system places insufficient emphasis on financial incentives and supervisory evaluation, which may reduce the enthusiasm and enforceability of policy execution, thereby impacting the effectiveness of the policy.

The overall application of demand-side policy tools is relatively low, with particularly insufficient use of government procurement (3/42), public-private partnerships (2/42), international exchange (1/42), pilot projects (10/42), and public charity (5/42), which together account for only 7.22% (21/291) of the total policy tools. Among them, the medical insurance tool has a higher usage frequency, accounting for 50% (21/42) of demand-side tools, reflecting policymakers' emphasis on alleviating the medical burden of NCD patients. However, the lack of demand-side policy tools such as government procurement and international exchange hinders the effective participation of market and social forces. This may result in insufficient public service provision and inadequate protection for vulnerable groups, thereby reducing the driving effect of demand-side policy tools on NCD prevention and control.

#### 3.2.3 Analysis of policy effectiveness

**Figure 6:**
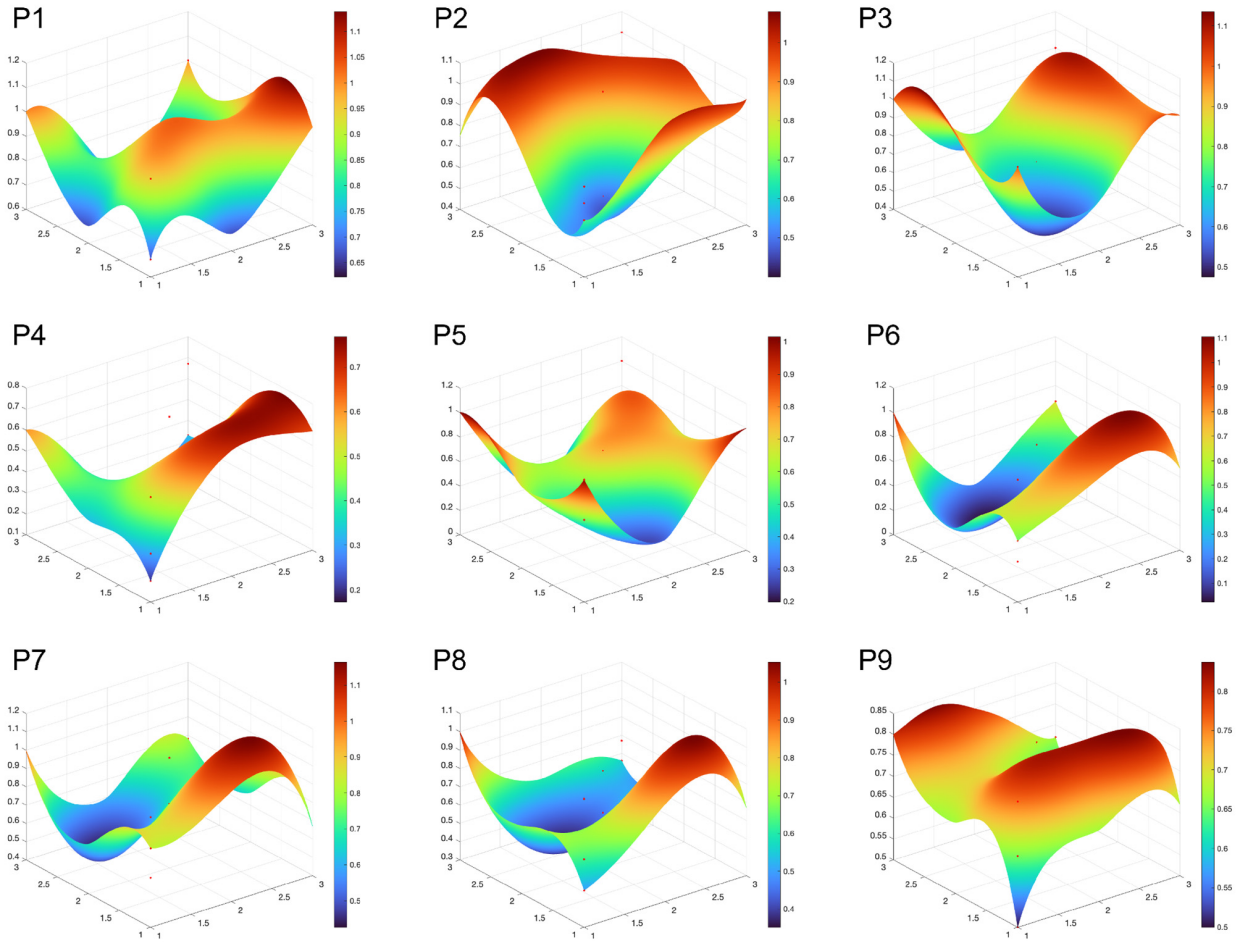
PMC surface plot of NCD policies. the lower the degree of convexity and concavity in the figure, the higher the level of policy consistency, indicating better coordination and stability between different factors in the policy.

Based on the PMC-Index modeling and calculation procedures described in the methodology section, this study conducted a quantitative analysis of nine NCD policy documents and assessed their effectiveness using the evaluation criteria outlined in Table 6. The results indicate that among the nine policy documents, four were rated as “Excellent” (P3, P7, P2, P5), while five were rated as “Acceptable” (P8, P9, P6, P1, P4) (Table 7). None of the documents were classified as “Perfect” or “Poor.” Policy P3 achieved the highest PMC score of 7.91, whereas policy P4 received the lowest score of 4.2. The average PMC score across the nine NCD policies was 6.19, falling within the “Excellent” range, suggesting that the overall design of China's NCD policy documents is relatively scientific and rational, with a high level of internal consistency and strong content effectiveness. In addition, the PMC surface plot generated from the model outputs revealed considerable variation among the policies. While the overall design is comprehensive, there remains significant room for improvement in the internal consistency and completeness across individual policies (Figure 6).

**Table 7:** PMC index evaluation of several of the policy texts.

	**P1**	**P2**	**P3**	**P4**	**P5**	**P6**	**P7**	**P8**	**P9**	**Mean**
X1	0.67	0.67	1	0.2	1	0.5	0.83	0.5	0.5	0.65
X2	0.67	1	0.67	0.33	0.67	0.33	0.67	0.67	0.67	0.63
X3	1	1	1	0.6	1	1	1	1	0.8	0.93
X4	0.67	0.83	1	0.67	1	1	1	0.83	0.67	0.85
X5	0.25	0.75	1	0.5	0.75	0.5	0.75	0.5	0.75	0.64
X6	0.6	0.5	0.7	0.7	0.3	0.8	1	0.6	0.8	0.67
X7	0.67	0.67	1	0.67	1	0.67	0.67	0.67	0.67	0.74
X8	0.4	0.4	0.6	0.2	0.2	0.2	0.6	0.6	0.2	0.38
X9	0.67	0.89	1	0.33	0.67	0.67	0.78	0.56	0.67	0.69
PMC	5.6	6.71	7.97	4.2	6.59	5.67	7.3	5.93	5.73	6.19
Rank	8	3	1	9	4	7	2	5	6	/
Rating	Acceptable	Excellent	Excellent	Acceptable	Excellent	Acceptable	Excellent	Acceptable	Acceptable	Excellent

**Figure 7:**
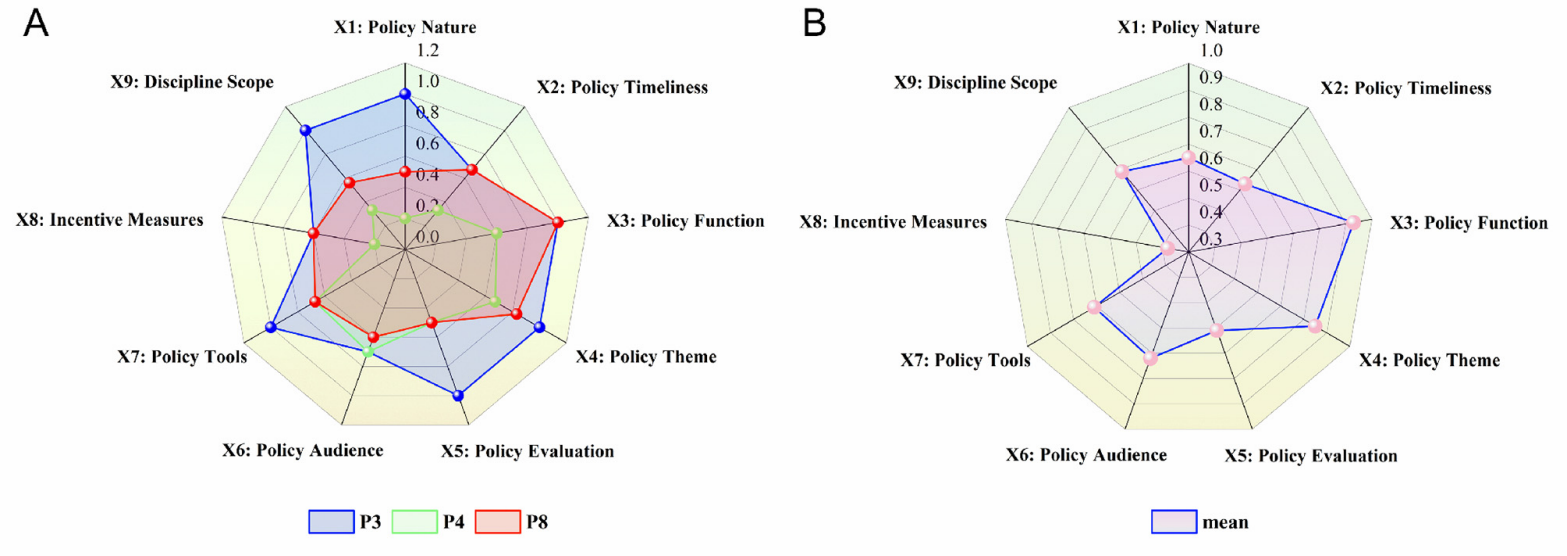
**(A)** Primary variable scores for P3, P4, and P8; **(B)** Average scores of primary variables in NCD policies.

Figure 7A visually presents the scores of the primary variables for the NCD policies with the highest (P3, 7.97), lowest (P4, 4.2), and middle (P8, 5.93) PMC index scores, allowing us to clearly observe the significant differences between policy texts. According to Table 7, the average scores of the nine primary variables in the NCD policy PMC index model are ranked from highest to lowest as follows: X3 > X4 > X7 > X9 > X6 > X1 > X5 > X2 > X8. Figure 7B reflects that there is still significant room for improvement in several aspects of China's NCD policy, including X1 policy nature, X2 policy timeliness, X8 incentive measures, and X9 related disciplines. Specifically:

1. The average scores of X3 Policy Function and X4 Policy Theme were 0.93 and 0.85, respectively, making them the highest scoring indicators among the nine. This reflects China's multidimensional consideration of policy functions and the systematic and comprehensive design of its NCD policies. In particular, X4 Policy Theme, as a core indicator of policy consistency, scored relatively high, indicating that China's NCD policies are broad in scope, covering areas such as healthcare reform and chronic disease prevention, with particular attention to the construction of primary healthcare systems and integrated disease management.
2. Although X7 Policy Instruments scored relatively high overall with an average score of 0.74, seven of the policies scored 0.67, which is below the average. This is because these policies lacked demand-oriented policy instruments. The role of demand-oriented policies in chronic disease governance is to align policies with the needs of the public and patients, utilizing various forms of social and market collaboration, such as government procurement, corporate partnerships, and international exchanges. These instruments improve the efficiency and accuracy of public health resource allocation, optimize the chronic disease diagnosis and treatment system, meet the needs of different population groups, and maximize the driving force of demand-oriented policies.
3. The average scores for X9 Involved Disciplines and X6 Policy Audience were 0.69 and 0.67, respectively. The main factor influencing the score for X9 was the relatively narrow range of disciplines covered by some policies. Given that chronic disease etiology involves multiple dimensions, including biological, behavioral, psychological, environmental, and social factors, as well as its long-term and complex nature, there is a need for an interdisciplinary approach in chronic disease policies. Effectively integrating the relevant fields in chronic disease management can not only promote interdisciplinary communication and cooperation, providing new perspectives and ideas for prevention and treatment, but also enhance the effectiveness of policy coordination and governance. In the NCD policies analyzed in this study, most policies covered public health, medicine, health economics, and nutrition, but there was insufficient focus on psychology, sociology, environmental science, and law.
4. The average score for X5 Policy Audience was 0.67, which, according to the evaluation results, aligns with the government's proposed “government-led, multi-departmental collaboration, mobilization of society as a whole, and full participation” approach to chronic disease governance. However, the evaluation results indicate that the policy pays insufficient attention to special groups such as farmers, the elderly, and people with disabilities. The primary reason for this may be that the Chinese government categorizes these groups as impoverished populations, making it difficult to clearly define the policy recipients and effectively safeguard their rights and interests. It is particularly concerning that five of the nine policies did not mention the involvement of social organizations, which limits the ability of societal forces to effectively contribute to chronic disease governance. The average score for X1 Policy Nature was 0.65, with P4, P6, P8, and P9 falling below the average. Overall, most policies are primarily regulatory, guiding, and advisory in nature, but they lack predictive and diagnostic features. Predictive policies help by forecasting the development of chronic disease patterns, enabling timely interventions and the efficient allocation of resources, avoiding waste of public health resources, and improving the precision and sustainability of policy governance. Diagnostic elements in policy design are essential starting points, especially for chronic diseases with such complexity. Only through a scientific evidence-based decision-making process can the key issues be identified, enabling targeted efforts in chronic disease prevention and control. The average score for X5 Policy Evaluation was 0.64. The relatively low score for this indicator is due to the lack of detailed policy content, unclear designation of responsible entities, and ambiguous implementation pathways, which can lead to policy execution deviations, wasting resources, and negatively impacting the effectiveness of policy implementation. The average score for X2 Policy Timeliness was 0.63, with the score being affected by the lack of long-term planning in some policies. However, overall, China's NCD policies have developed a governance system that includes long-term planning, mid-term coordination and improvement, and short-term precision execution.
5. X8 Incentive Measures received the lowest score among the nine indicators (0.38). Talent is fundamental to chronic disease prevention and control. Among the nine policies, only P7 includes policies related to talent incentives. The absence of such content can lead to the loss of skilled professionals in chronic disease management, impacting the long-term effectiveness of policy implementation. None of the nine policies mention tax incentives or government subsidies, which makes it difficult to motivate enterprises and other social forces to actively participate in chronic disease governance. The lack of incentive and constraint mechanisms makes it difficult to activate market participation in chronic disease governance, directly affecting the long-term effectiveness and sustainable development of NCD policy implementation.

### 3.3 NCD governance policies analysis

In response to the growing global burden of non-communicable diseases (NCDs), governments worldwide have introduced a range of policies aimed at reducing the associated public health and economic pressures, thereby fostering a supportive environment for effective NCD governance. Although China has made notable progress in NCD prevention and control, it still lags behind many developed countries in terms of governance depth and comprehensiveness-largely due to its relatively late policy initiation and regional disparities in development.

To better understand the similarities and differences in national NCD policy frameworks and implementation outcomes, this study systematically compares the governance approaches of China, the United States, Australia, and Japan (see Table 8). These countries were selected because they represent high-income nations with mature healthcare systems and established NCD strategies. The comparative analysis identifies distinct governance models, highlights best practices, and offers valuable insights to inform the optimization of China's NCD policy system.

**Table 8:** International comparison of NCD policies.

**Country**	**Representative policies**	**Governance strategies**	**Management entities**	**Policy outcomes**
China	“Healthy China 2030 Planning outline” (2016), “China's Medium- and long-term plan for the prevention and control of NCDs (2017–2025)” (2017)	Strengthen early screening and intervention for NCDs, promote the transition from disease treatment to health management, and conduct nationwide health education and health promotion activities.	National Health Commission, local governments at all levels, medical institutions, community health service centers	The overall effectiveness of NCD management is acceptable, with progress in health education and early screening. However, disparities in medical resources and management systems across regions have led to uneven outcomes.
United States	“Healthy People 2020” (2010), “Healthy People 2030” (2020)	Improve NCD management through various measures such as epidemiological surveillance, policy and environmental interventions, healthcare system interventions, and community programs.	Department of Health and Human Services (HHS), Centers for Disease Control and Prevention (CDC), state health departments, community organizations	The U.S. has shown outstanding performance in medical technology innovation, with improvements in patients' self-management abilities, but high healthcare costs and persistent health disparities remain.
Australia	National Strategic Framework for Chronic Disease (NSFCC) (2017), “Australia's Health Strategy” (2018)	Implement general practitioner-led Comprehensive Primary Health Care (CPMP) and Team Care Arrangements (TCAs), and strengthen health performance monitoring.	Federal government, state and territory governments, Department of Health and Aged Care, Australian Institute of Health and Welfare, general practitioners, community health service centers	After policy implementation, care costs were reduced, health promotion effects were significant, and chronic disease management achieved good results through collaboration with general practitioners.
Japan	“Specific Health Checkups and Health Guidance” (2008), “Healthy Japan 21 (Second Phase)” (2013)	Strengthen specific health checkups and health guidance, implement early screening and intervention, promote mandatory universal health insurance system, and optimize the integration of medical and nursing services.	Ministry of Health, Labor and Welfare, health insurance organizations, community health service centers	Early screening and intervention have shown significant effects, integration of medical and nursing services has been highly effective, and positive progress has been made in addressing the chronic disease challenges brought by population aging.

#### 3.3.1 Governance strategies

China's NCD policy emphasizes “co-building and sharing” and “prevention first” focusing on the construction of the primary healthcare service system and advocating the concept that “everyone is their own first responsibility.” At the same time, the policy strengthens health education and promotes healthy lifestyles, covering various aspects such as healthcare services, health equity, and social security. The policy's goal is to reduce the impact of NCDs on public health through systematic measures, lower premature mortality caused by NCDs, improve the overall health level of residents, and achieve population-wide, full-cycle health management. The United States' NCD policy focuses on cross-sector, multi-stakeholder, and multi-dimensional comprehensive interventions aimed at providing a favorable health management environment for citizens. This policy not only seeks to reduce healthcare costs but also enhances citizens' health literacy, with particular emphasis on the application of technology in NCD governance, promoting health equity, and strengthening individual self-management capabilities (70). Australia's NCD policy emphasizes early screening and timely intervention, with a general practitioner-led comprehensive service system covering full-cycle management of patients, cross-sector collaboration, social support, health information sharing, and the application of digital health technologies. The policy also emphasizes long-term care for NCDs, community interventions, and health promotion (71). Japan's NCD policy promotes early intervention through early screening and health guidance, with more detailed NCD prevention and control measures targeting aging-related issues. The policy emphasizes the integration of medical care and nursing services, ensuring that patients receive continuous, high-quality healthcare. Additionally, the Japanese government places particular importance on health education, encouraging citizens to adopt healthy eating habits and engage in moderate physical activity to reduce the incidence of NCDs. The policy also focuses on community participation and ensures equal access to public health services for all citizens through mandatory universal health insurance, thereby reducing the financial burden on patients (72).

#### 3.3.2 Management entities

China's NCD governance system is led by the National Health Commission, adopting a top-down governance model. The National Health Commission is responsible for formulating national-level NCD policies, while local governments develop and implement specific measures based on central policies. Medical structures and community health service centers play a crucial role in this system, directly providing basic healthcare services to citizens, including health education, early screening, disease diagnosis, and management. In the United States, NCD policies are guided and regulated by the Department of Health and Human Services (HHS), with the Centers for Disease Control and Prevention (CDC) as its subsidiary, responsible for monitoring, data collection, and formulating health intervention measures for NCDs. The U.S. emphasizes the responsibilities and rights of state governments in NCD governance, with state and local health departments formulating and implementing NCD policies based on local conditions. Japan's NCD governance adopts a horizontal collaborative governance model involving national and local governments, as well as social organizations such as health insurance institutions (73). The Ministry of Health, Labor and Welfare leads the formulation and promotion of policies nationwide, while health insurance institutions provide health management services for NCD patients within the health insurance system. Local governments and community health service centers collaborate to implement health checkups and health guidance programs, particularly conducting regular screenings and interventions for high-risk groups (74).

#### 3.3.3 Policy outcomes

The Chinese government adheres to a people-centered approach in NCD governance, actively promoting the integration of health into various policies, especially under the framework of the Healthy China strategy. To alleviate the burden of NCDs and improve public health, China has introduced a series of national policies, laws, and regulations. With the continuous improvement of the NCD policy system, the capacity and level of comprehensive prevention and control have gradually increased, and the policy practice has achieved significant results. Since the launch of the National NCD Prevention and Control Zones initiative in 2010, by 2023, a total of 488 national-level NCD prevention and control zones have been established, with a county-level coverage rate exceeding 17%. Additionally, 2,880 counties have launched the National Healthy Lifestyle Campaign, with a coverage rate of 97.3%. Citizens' health literacy has increased from 8.8% in 2012 to 27.8% in 2022 (75). The premature mortality rate from major NCDs has decreased from 18.5% in 2015 to 15.0% in 2023. By 2024, the average life expectancy in China had reached 79 years, an increase of 5.95 years compared to 2009. According to the analysis of data from the China Chronic Disease and Risk Factor Surveillance (CCDRFS) by Professor Wang Limin's research team, the standardized prevalence rate of hypertension among adults aged 18-69 decreased from 29.6% in 2010 to 24.7% in 2018. However, the treatment and control rates for hypertension remain low, and the primary healthcare system faces significant pressure and challenges (76).

At the same time, the lack of scientific evidence, the continuous increase in the burden of non-communicable diseases, regional disparities in NCD governance service levels, and the collaborative inertia in multi-sectoral cooperation remain major challenges that urgently need to be addressed in China's NCD governance (9).

The United States' NCD management policy emphasizes cross-sector, multi-stakeholder cooperation to promote NCD prevention, early screening, and treatment and management. Under the health policy framework centered on the Healthy People initiative, the U.S. has achieved significant accomplishments, including: reducing major causes of death, such as heart disease and cancer; increasing preventive behaviors, such as childhood vaccination; and reducing risk factors such as smoking, hypertension, and high cholesterol (77). The self-management education program for NCD patients is a key component, covering 56.4% of U.S. counties. However, it is worth noting that 80.4% of the workshops and 82.1% of the registered participants are concentrated in metropolitan areas, with a significant disparity in access to medical services and self-management education between rural and urban areas. In terms of the application of digital health technologies, the U.S. has also made significant progress. For example, remote patient monitoring (RPM) has been significantly expanded and integrated into routine clinical care, with legislative support. Especially during the COVID-19 pandemic, the application of RPM technology saw exponential growth, not only meeting the medical needs of NCD patients but also greatly reducing the burden on the U.S. public health system (78). Nevertheless, the United States still faces significant challenges in NCD governance. NCDs account for seven of the top ten causes of death in the U.S., and six out of ten Americans suffer from at least one NCD, placing a heavy economic burden on the healthcare system. NCD-related healthcare spending accounts for 90% of total healthcare expenditure, and this proportion is expected to continue rising (79).

Australia's primary healthcare system has played a crucial role in NCD governance. With the development of this system, Australia's average life expectancy has reached a historic high. The life expectancy for males born in 2023 is 81.3 years, and for females, it is 85.1 years. The premature mortality rate from chronic diseases such as cardiovascular diseases has significantly decreased, especially since the implementation of the national cervical screening program, which has halved the number of deaths from cervical cancer. The general practitioner-led Medicare program plays a central role in the prevention, screening, diagnosis, and management of NCDs. According to data, during the 2022-2023 period, approximately 60% of patients visiting general practitioners (10.2 million) had long-term health issues, with 16% (4.1 million) receiving chronic disease management services. This highlights the central role of general practitioners in Australia's NCD governance system (80). Australia's NCD policy focuses on prevention and planned multidisciplinary care, rather than emergency care, which aligns with the World Health Organization's policy philosophy. Compared to other Organization for Economic Co-operation and Development (OECD) member countries, Australia's primary healthcare services are more accessible. However, Indigenous Australians, low-income groups, and residents in rural areas still face significant challenges in accessing self-management education and health services, with the burden of NCDs being particularly significant in these groups (81).

Since the implementation of the specific health checkups and health guidance policy, Japan has made significant progress in NCD prevention and management. Japan has made substantial strides in improving population health and is one of the leading countries globally in life expectancy. The life expectancy for those born in 2021 is 85.2 years, an increase of 5.8 years compared to 1990. The mortality rate from major non-communicable diseases has decreased, although the rate of decline has slowed (82). Additionally, Japan has achieved universal health insurance coverage, ensuring that NCD patients can continuously receive healthcare services, thus reducing their financial burden. The government has also made significant progress in advocating for healthy eating. Studies have shown that the mortality rate from brain hemorrhage has significantly decreased, partly due to the health benefits of Japanese diets and traditional foods, reflecting the effectiveness of the government's health lifestyle promotion and the success of its NCD policies. Looking ahead, the Japanese government will need to improve the level and quality of NCD services in the context of aging, particularly as the gap in healthcare resources between urban and rural areas remains an urgent issue that needs to be addressed (83).

Through a systematic comparative analysis of the NCD policies of China, the United States, Australia, and Japan, this study finds that: China's NCD policy emphasizes “co-building and sharing” and “prevention first,” focusing on the construction of the primary healthcare service system and improving public health literacy through health education and lifestyle promotion. The United States, on the other hand, employs cross-sector, multi-stakeholder, and multi-dimensional interventions to promote early screening and self-management of NCDs, while also strengthening health equity and the application of technology in governance. Australia's policy focuses on early screening, general practitioner-led NCD management, and multidisciplinary care, emphasizing long-term care and community intervention for NCDs. Japan, in contrast, integrates medical and nursing care, early intervention, and health guidance, providing effective NCD management, especially for the elderly, in the context of aging.

## 4 Discussion

The innovation of this study lies in balancing the subjectivity of qualitative analysis and the objectivity of quantitative analysis through complementary research methods. This provides a richer research perspective, broader scope, and emphasizes practicality. However, this study still has certain limitations, including: (1) Despite the extensive and locally distinctive policies implemented by local governments in China for chronic disease prevention and control, this study did not include local policies in the sample analysis. This limitation may result in the research findings not fully reflecting the overall situation of chronic disease policies in China, particularly the specific role and effectiveness of local policies in chronic disease prevention and control, as well as their coordination and interaction with national-level policies. (2) Insufficient analysis of the synergies between policy tools: Although this study points out the imbalance in the use of policy tools, the analysis of the interactions and synergies between different policy tools remains insufficient. In particular, there is a lack of in-depth discussion on how policy tools can work together to enhance policy effectiveness and social participation. (3) Insufficient long-term evaluation of policy effectiveness: The PMC model used in this study primarily assesses the internal consistency of policy texts but does not fully consider the actual effects after policy implementation. To address the limitations of the current study, future research could focus on the following areas: (1) Future studies could further reveal the unique roles and interactions of policies at different levels in NCD prevention by conducting a layered comparison between local and national policies. (2) Empirical methods, such as difference-in-differences (DID), can be applied to analyze the impact of policy implementation on long-term indicators such as the incidence rate, mortality rate, and healthcare costs of NCDs, providing more reliable data support for policy evaluation. (3) In-depth research on the synergies between different types of policy tools (supply-side, demand-side, and environmental) can help explore how to optimize tool combinations to enhance policy effectiveness. (4) Leveraging new technologies such as artificial intelligence, big data, and large language models can improve the efficiency and accuracy of policy text analysis, extracting deeper policy insights and trends. (5) Long-term tracking of the implementation effects of NCD policies should be conducted to assess their impact on public health, healthcare burden, and social costs, providing a basis for policy optimization.

## 5 Conclusion

This study combined qualitative and quantitative research methods to conduct a multidimensional systematic analysis of China's NCD policies from 2009 to 2025. The findings effectively address the three questions raised by this study:

Question 1: What are the themes of China's NCD policies? In terms of policy themes, this study identified and summarized six major themes in China's NCD policies using LDA topic modeling based on 50 policy samples. These themes include primary healthcare, health promotion, health equity, healthcare reform and regulation, chronic disease prevention and control, and public health governance. The findings align with the Chinese government's priorities in NCD governance. First, improving the level of primary healthcare services has always been a key focus of the government. In 2025, the National Health Commission issued the “Opinions on Optimizing the Layout and Construction of Primary Medical and Health Institutions,” particularly focusing on improving healthcare systems in economically underdeveloped areas such as rural and township regions. The policy set the goal of achieving full coverage of basic medical and health services in rural and community areas by 2027, with everyone being able to reach the nearest healthcare station within 15 minutes. Furthermore, to effectively bridge the healthcare service gap between regions and between urban and rural areas and promote health equity (84, 85), the Chinese government continues to improve the medical insurance system (86), and promotes national fitness campaigns to enhance public health and safeguard residents' basic health rights.

Question 2: Are the policy instruments used in China's NCD policies reasonable? In terms of policy instruments, this study applied policy instrument theory and used NVivo 15 software to conduct content analysis on 50 NCD policy samples. The results reveal that, overall, the use of policy instruments in China's NCD policies exhibits structural imbalances and internal inconsistencies. Policy makers tend to favor the use of supply-oriented (49.1%) and environment-oriented (36.4%) policy instruments, which provide a solid external environment and basic support for the development of NCD policies. However, the use of demand-oriented policy instruments is significantly lacking (14.4%), which is consistent with the findings of existing research (8). Specifically, among supply-oriented policies, public services and infrastructure development are used more frequently, while talent development, financial investment, and standardization are less frequently utilized. Within environment-oriented instruments, internal inconsistency is more pronounced. Strategic measures dominate the use of environment-oriented instruments, while financial and tax policy tools, which could encourage the participation of enterprises, social organizations, and other societal forces in chronic disease management, are used much less. Among demand-oriented policy instruments, except for medical insurance, government procurement, public-private partnerships, international cooperation, public philanthropy, and pilot programs are relatively underused.

Question 3: Are China's NCD policies effective? In terms of policy effectiveness, this study applied the PMC-Index model to analyze nine policy samples. The results revealed that the average PMC score of the nine NCD policies was 6.16, with an evaluation rating of “Excellent.” Among the nine policy samples, four were rated as “Excellent” and five as “Acceptable.” No policy samples were rated as “Perfect” or “Poor.” This suggests that China's NCD policies are generally well-designed and that their effectiveness is relatively high. However, significant differences still exist between policies, and aspects such as X1 Policy Nature, X2 Policy Timeliness, X8 Incentive Measures, and X9 Involved Disciplines should be prioritized for improvement.

Additionally, through an analysis of the external characteristics of the policy texts, it was found that the collaboration among decision-making bodies is weak in the design and issuance of NCD policies, with policies often being issued by single departments. Therefore, future NCD policies will require more cross-departmental collaborative design and governance.

## 6 Policy implications

As the global burden of NCDs increases, scientific and comprehensive policy planning is crucial for achieving universal health coverage. The effectiveness of policy planning directly impacts the scope and quality of NCD prevention, treatment, and management, thus affecting the realization of universal health. According to the Policy Triangle Theory, the success of a policy depends not only on the design of its content but also on the interaction between the policy environment, policy content, and policy process (87, 88).

### 6.1 Policy environment

The construction of the policy environment is crucial for NCD governance and universal health coverage. The complexity of NCDs requires cross-departmental, cross-disciplinary, and cross-regional cooperation. In this context, coordination and collaboration among policy actors are essential. (1) The complexity of NCD governance makes it impossible for a single department to respond effectively. Therefore, policy planning should promote cross-departmental collaboration, covering sectors such as health, finance, education, and social security. Government agencies should collaborate with public health management departments, hospitals, research institutions, non-governmental organizations (NGOs), and NCD patients, among other stakeholders, in policy formulation and implementation. This ensures that resources and efforts from different departments and sectors are coordinated, thus enhancing the effectiveness and implementation of policies. (2) In the policy environment, the application of digital health technologies (such as artificial intelligence, big data, natural language processing, etc.) should be promoted to enhance the scientific basis and accuracy of policy decisions. By leveraging technological tools, not only can the effectiveness of decisions be improved, but the flow and sharing of information among stakeholders can also be ensured, enhancing the efficiency of policy implementation. (3) Emphasis should be placed on the foresight of the policy environment. Policy formulation needs to anticipate future trends in NCD development and consider the coordination of long-term and short-term goals from the early stages of policy planning. Regular dynamic evaluations of policy effects should be conducted to ensure that policies are adjusted in a timely manner based on changing environments and needs.

### 6.2 Policy content

The design of policy content determines the specific measures and direction of NCD governance, directly influencing resource allocation and the achievement of policy goals. (1) Policy content should place greater emphasis on the use of demand-driven policy tools, encouraging the government to guide social forces in participating in NCD governance through mechanisms such as service purchasing and public-private partnerships. These tools effectively mobilize resources from all sectors of society, improving the accessibility and equity of NCD treatment and management services. (2) Health education is a key component of policy content. Particularly in NCD prevention and control, public health education should be strengthened to raise awareness of NCDs and promote the idea that “everyone is primarily responsible for their own health.” In addition, policies should guide the broader participation of society in adopting healthy lifestyles, thereby improving overall public health. (3) Policies should strengthen fiscal and tax incentives to encourage social forces to participate in NCD governance, increasing investment from both the public and private sectors in NCD-related medical services. Tax policies and financial subsidies can be used to support health coverage for low-income and specific social groups, ensuring equitable access to health protection for all populations. (4) The formulation of policy content should take local conditions into account, encouraging research focused on local populations to ensure that research results are locally applicable and provide scientific evidence for NCD prevention and control. At the same time, policy design should consider the coordination and complementarity between local and national policies to achieve coordinated governance across the country.

### 6.3 Policy process

The policy process determines the implementation and evaluation of the policy and is key to ensuring the achievement of policy goals. (1) Stakeholder participation is crucial in the policy formulation, implementation, and evaluation processes. The policy process should involve multiple stakeholders, including government departments, public health management departments, healthcare providers, patient groups, and research institutions. The broad participation of stakeholders ensures that the policy content is more closely aligned with actual needs, enhancing its operability and fairness. (2) To ensure the fairness and transparency of policy implementation, a clear policy evaluation system should be established. Policy evaluation should consider not only the short-term effects of policy implementation but also long-term health indicators, such as the incidence and mortality rates of NCDs, as well as healthcare costs. An independent third-party evaluation mechanism should be introduced during the policy process to ensure the fairness and objectivity of the evaluation results. (3) Accountability must be strengthened during policy implementation. The responsible parties for policy implementation should be clearly defined, and any actions that fail to comply with regulations or meet established goals should be held accountable. To ensure the effectiveness of accountability, regular performance evaluations should be conducted, and incentive and penalty mechanisms should be established to encourage stakeholders to fulfill their responsibilities, ensuring that policy goals are successfully achieved. (4) A real-time feedback mechanism should be established during policy execution. Feedback obtained during policy execution should be promptly incorporated into the evaluation system, allowing the policy to be dynamically adjusted based on actual outcomes. This ensures that the policy can make necessary adjustments at different stages based on actual circumstances, thereby enhancing its sustainability and adaptability.

## Data Availability

The original contributions presented in the study are included in the article/supplementary material, further inquiries can be directed to the corresponding author.
